# Early-cleavage is a reliable predictor for embryo implantation in the GnRH agonist protocols but not in the GnRH antagonist protocols

**DOI:** 10.1186/1477-7827-7-20

**Published:** 2009-03-03

**Authors:** Wen-Jui Yang, Yuh-Ming Hwu, Robert Kuo-kuang Lee, Sheng-Hsiang Li, Steven Fleming

**Affiliations:** 1 Department of Obstetrics and Gynecology, Mackay Memorial Hospital, Taipei, Taiwan; 2 Mackay Medicine, Nursing and Management College, Taipei, Taiwan; 3 Department of Medical Research, Mackay Memorial Hospital, Taipei, Taiwan; 4 Department of Obstetrics & Gynecology, University of Sydney, Westmead Hospital, NSW 2145, Australia; 5 Department of Obstetrics and Gynecology, Ton-Yen General Hospital, Hsinchu, Taiwan

## Abstract

**Background:**

To test if early-cleavage was a strong predictor of pregnancy in patients receiving either a GnRH agonist long protocol or a GnRH antagonist protocol for in-vitro fertilization treatment (IVF) and intracytoplasmic sperm injection (ICSI).

**Methods:**

This retrospective study included 534 patients undergoing a fresh cycle of oocyte retrieval and the day-3 embryo transfer (from 22 to 46 years old). Of the 534 patients treated, 331 received a GnRH agonist long stimulation protocol (GnRH agonist group) for ovarian stimulation and 203 patients received a GnRH antagonist protocol (GnRH antagonist group). In each group, patients who had at least one early-cleavage embryo transferred were designated as the 'early-cleavage' subgroup. Patients who had no early-cleavage embryos transferred were designated as the 'late-cleavage' subgroup.

**Results:**

The early cleavage rate was significantly lower in the GnRH antagonist group compared with that in the GnRH agonist group (IVF cycles: 34% versus 20%; ICSI cycles: 50% versus 37.8%, respectively, P < 0.0001). In the GnRH agonist group, the pregnancy rates were significantly higher in the early-cleavage subgroup than those in the late-cleavage subgroup (53.7% vs 33.9%, *P *< 0.0001). In the GnRH antagonist group, the pregnancy rates were not significantly different between the early-cleavage and late-cleavage subgroups (45.9% vs 43.8%, P > 0.05).

**Conclusion:**

Early cleavage of zygote is not a reliable predictor for embryo implantation potential in using the GnRH antagonist protocol. Furthermore, the implantation rates between the GnRH agonist and GnRH antagonist groups were comparable.

## Background

In order to reduce multiple pregnancies and achieve a maximal rate of implantation, selection of the most viable embryo for transfer has become a high priority in assisted reproduction treatment. Traditionally, embryo selection is performed by using embryo morphology as the guideline. Other additional selection methods include oocyte and zygote morphology, blastomere symmetry and blastocyst culture. Recently, observation of embryonic first mitosis has been emphasized. Several studies have shown that embryonic early cleavage, which occurs at 25–27 hours post insemination for *in vitro *fertilization (IVF)/intracytoplasmic sperm injection (ICSI), can be an additional indicator of viable embryos [1-6]. Most of these previous studies were only using the gonadotrophin-releasing hormone (GnRH) agonist long protocol for pituitary suppression.

Recently, a GnRH antagonist protocol has become available in assisted reproduction therapy. The advantages of GnRH antagonist are associated with a lower consumption of gonadotropins, a shorter duration of stimulation, a lower risk of ovarian hyperstimulation syndrome (OHSS) [7], and a lower cancellation rate, especially in poor responders [8-10]. Previous studies have shown that using the GnRH antagonist protocol had the comparable pregnancy rate compared with the GnRH long agonist protocol [8-10]. However, some studies have shown that GnRH receptors are expressed in human and mouse preimplantation embryos [11,12], and addition of GnRH antagonist to mouse embryo culture media inhibits preimplantation embryo development [11]. We wondered whether the effects of these two different protocols upon embryonic development were the same. In our previous study, we had found that early-cleavage is not a reliable predictor for embryo implantation in women over 35 years old using the GnRH antagonist protocol [13]. In this study, we recruited women without age limitation to investigate if early-cleavage is a good predictor for their pregnancy under the treatment of GnRH antagonists. Therefore, the aim of this study was to compare the embryonic early-cleavage rates between the GnRH agonist and GnRH antagonist groups and to investigate if early-cleavage was a good predictor for IVF outcome in both groups.

## Methods

### Study subjects

This study was a retrospective study of IVF/ICSI outcome based on the medical records of patients in the Fertility Unit of MacKay Memorial Hospital from the time period, January 2004 to December 2006. The study protocol was approved by the Institutional Review Board of MacKay Memorial Hospital in Taipei, Taiwan.

### Ovarian stimulation protocols

Two stimulation protocols were used in this study; the GnRH agonist long protocol (GnRH agonist group) and the GnRH antagonist protocol (GnRH antagonist group). In the GnRH agonist group, leuprolide acetate (Takeda Pharma GmbH, Stolberg, Germany) was given at a daily dose of 1 mg, starting on day 21 of the previous cycle. Once serum levels of E_2 _< 30 pg/ml were achieved, the daily dose of leuprolide acetate was reduced to 0.5 mg. Recombinant follicle-stimulating hormone (FSH; Gonal-F; Serono Laboratories, Aubonne, Switzerland) and human menopausal gonadotropin (hMG; Menopur; Ferring GmbH, Kiel, Germany) were given until the day of hCG administration. The doses were adjusted according to the patient's ovarian response. In the GnRH antagonist group, recombinant FSH and hMG were administrated daily from the second day of the menstrual cycle. The doses were also adjusted according to the patient's individual ovarian response. Once the dominant follicle reached 14 mm in mean diameter, cetrorelix (Cetrotide; Serono, Baxter Oncology GmbH, Halle, Germany) was administered subcutaneously at a dose of 0.25 mg daily until the day of hCG administration.

In both groups, 10,000 IU hCG (Profasi; Serono Laboratories, Aubonne, Switzerland) was administered when at least two follicles reached 18 mm in diameter, and oocyte retrieval was performed 34–36 hours later. Upon completion of oocyte collection and IVF/ICSI, embryos were graded morphologically by the same embryologist. Embryo transfer was performed 72 hours after the oocyte retrieval. In each stimulation protocol, patients who had at least one early-cleavage embryo transferred were designated as the 'early-cleavage' subgroup. Patients who had no early-cleavage embryos transferred were designated as the 'late-cleavage' subgroup.

The luteal phase was supported by vaginal supplementation of 200 mg micronized natural progesterone (Progeffik; Effik, Paris, France) three times a day. To assess treatment outcome, serum hCG was measured 14 days after oocyte retrieval. An increase of serum hCG to levels above 50 IU/l indicated pregnancy.

To minimize confounding factors, patients diagnosed with polycystic ovaries, a severely poor response (fewer than two retrieved oocytes and an E_2 _level ≤ 300 pg/ml on the day of human chorionic gonadotrophin (hCG) administration), and cycles for oocyte donation were excluded from the study. Only patients undergoing their first stimulated cycle were included and only one treatment cycle per patient was analyzed. A total of 534 embryo transfer cycles (from 22 to 46 years old) were included in this study. There were 331 cycles treated with an agonist protocol (192 cycles from IVF while 139 cycles for ICSI) and the other 203 cycles treated with an antagonist protocol (116 cycles from IVF while 87 cycles from ICSI). Patients with male factor infertility (which was defined by the presence of any of the following parameters: sperm concentration < 20 × 10^6^/ml, motility < 50%, and normal morphology < 15%) were treated with ICSI, while others without male factor infertility were treated with conventional insemination for IVF.

### Insemination and ICSI

Conventional IVF or ICSI was performed 3–5 h after oocyte aspiration. For conventional IVF, each oocyte was inseminated with 20 × 10^3 ^motile spermatozoa in a single drop of 20 μl medium (Quinn's Advantage Fertilization medium; SAGE IVF Inc. Trumbull, Connecticut, U.S.A.). For the ICSI procedure, 1–2 μl washed spermatozoa were placed in 7% polyvinylpyrrolidone (PVP; SAGE IVF Inc.) and injected using standard techniques. Each embryo was cultured in a single drop of 20 μl medium (Quinn's Advantage Cleavage medium; SAGE IVF Inc.) supplemented with 10% synthetic serum substitute (Quinn's Advantage Serum Protein Substitute; SAGE IVF Inc.) covered with mineral oil (SAGE IVF Inc.) in an atmosphere of 5% O_2_, 5% CO_2 _and 90% N_2 _at 37°C.

### Assessment of fertilization, early cleavage and embryo quality

Normal fertilization was confirmed by the presence of two pronuclei and two polar bodies 16–20 h (day1) after insemination for IVF or ICSI. On the same day, early cleavage examination was performed 25–27 hours after insemination. Embryos displaying two cells at inspection were designated as 'early cleavage'. The embryos that had not yet cleaved to the 2-cell stage were designated as 'late cleavage'. Embryos were further examined for their quality at 44–46 hours (day 2) and at 66–68 hours (day 3). The day 2 and day 3 embryo scoring system (modified from Veeck [14]) was as follows: for day 2 embryos, namely: score 1: embryo with blastomeres of equal size and no cytoplasmic fragmentation; score 2: embryo with blastomeres of equal or unequal size and cytoplasmic fragmentation ≤ 10% of the embryo surface; score 3: embryo with blastomeres of equal or unequal size and 11%–49% overall cytoplasmic fragmentation; score 4: embryo with blastomeres of equal or unequal size and cytoplasmic fragmentation ≥ 50% of the embryo surface. For the day 3 embryo scoring system, this was the same as described above except score 2 denotes ≤20% cytoplasmic fragmentation and score 3 denotes 21%–49% cytoplasmic fragmentation.

### Statistical analysis

Statistical analysis was performed using SPSS software version 12.0 for Windows (Statistical Package for Social Sciences, Inc., USA). The differences of means between two variables were calculated using the Mann-Whitney *U *test. The differences in binary variables were calculated using the chi-square test. A *P *value of < 0.05 was considered statistically significant.

## Results

A total of 534 treatment cycles were analyzed. Of these, 331 cycles were from the agonist group (192 cycles from IVF and 139 cycles from ICSI) while 203 cycles were from the antagonist group (116 cycles from IVF and 87 cycles from ICSI). Cycle characteristics of the agonist and antagonist groups are given in Table 1. The percentages of good quality of embryos (grade 1 and grade 2 embryos) derived from IVF and ICSI were no significantly different in both agonist and antagonist groups. The mean duration of stimulation was significantly shorter in the GnRH antagonist group compared with that in the GnRH agonist group (9.9 ± 0.1 vs.11.3 ± 0.1, P < 0.05). The E2 levels on the day of hCG injection were significantly lower in the GnRH antagonist group than those in the GnRH agonist group (1731.5 ± 81.1 vs. 2347.8 ± 71.9, P < 0.0001). Furthermore, the early cleavage rate was significantly lower in the GnRH antagonist group compared with that in the GnRH agonist group (IVF cycles: 34% versus 20%; ICSI cycles:50% versus 37.8%, respectively, P < 0.0001).

**Table 1:** Comparisons of cycle characteristics in the agonist and the antagonist groups.

	Agonist group	Antagonist group	P value
No. of cycles	331	203	
			
ICSI rate	42% (139/331)	43% (87/203)	NS
Age of patients	33.9 ± 0.2 (22–45)	34.4 ± 0.3 (26–46)	NS
Duration of stimulation (days)	11.3 ± 0.1	9.9 ± 0.1	< 0.05
No. of ampoules used per cycle	38.6 ± 0.5	35.4 ± 0.4	NS
Peak E_2 _level	2347.8 ± 71.9	1731.5 ± 81.1	< 0.0001
No. of oocytes retrieved per cycle	10.0 ± 0.3	8.2 ± 0.4	< 0.05
No. of mature oocytes per cycle	9.0 ± 0.3	7.2 ± 0.3	< 0.05
No. of normal fertilized oocytes per cycle	6.1 ± 0.3 (2019)	5.7 ± 0.3 (1157)	NS
Total no. of embryos	2259	1166	
			
Good embryo rate			
IVF	54.6% (715/1309)^a^	56.5%(375/664)^c^	
ICSI	54.4% (517/950)^b^	56% (281/502)^d^	
No. of embryo transfer	3.8 ± 0.1	3.32 ± 0.9	NS
			
Embryonic early cleavage rate			
IVF	34% (450/1309)	20% (133/664)	< 0.0001
ICSI	50% (476/950)	37.8% (190/502)	< 0.0001
			
Pregnancy rate (%)			
IVF	46.9% (90/192)	44.8% (52/116)	NS
ICSI	46.8% (65/139)	44.8% (39/87)	NS
			
Implantation rate			
IVF	21.9% (160/730)	19% (73/384)	NS
ICSI	21.2% (112/528)	18.9% (55/290)	NS
			
On going pregnancy rate			
IVF	35.9% (69/192)	38.8% (45/116)	NS
ICSI	36% (50/139)	39% (34/87)	NS

Note: Values are the mean ± SEM. NS: no significance.

^a ^versus

^b^: NS.

^c^versus^d^: NS.

A total of 3425 embryos were analyzed; 2259 embryos were from the agonist group and 1166 embryos were from the antagonist group. In the agonist group, 926 (41%) of 2259 embryos were early-cleavage embryos, and the remaining 1333 (59%) embryos were late-cleavage embryos. There were significant differences in the day 2 and day 3 embryo scores and mean numbers of blastomeres between the early and late-cleavage embryo subgroups (Table 2). In the agonist group, 216 cycles had at least one early-cleavage embryo for transfer while the remaining 115 cycles had all late-cleavage embryos for transfer. The pregnancy rate was significantly higher in the early-cleavage subgroup then that in the late cleavage subgroup (53.7% vs. 33.9%, P < 0.0001) (Table 2).

**Table 2:** Embryo scores and mean numbers of blastomeres of the early-cleavage and late-cleavage subgroups in the agonist group.

	Early-cleavage subgroup	Late-cleavage subgroup	P value
Day 2 embryo score	1.8 ± 0.03	2.1 ± 0.03	< 0.0001
No. of day 2 blastomeres	4.0 ± 0.04	3.17 ± 0.04	< 0.0001
Day 3 embryo score	1.9 ± 0.03	2.5 ± 0.03	< 0.0001
No. of day 3 blastomeres	7.28 ± 0.06	6.16 ± 0.06	< 0.0001
Pregnancy rate (%)	116/216 (53.7)	39/115 (33.9)	< 0.0001

Note: Values are the mean ± SEM.

In the antagonist group, 323 (27.7%) of 1166 embryos were early-cleavage embryos while the remaining 843 (72.3%) embryos were late-cleavage embryos. There were no significant differences in the day 2 and day 3 embryo scores or in the day 3 numbers of blastomeres between the early and late-cleavage embryo subgroups in the antagonist group (1.92 ± 0.04 vs. 2.11 ± 0.04, 2.02 ± 0.06 vs. 2.10 ± 0.06, and 7.29 ± 0.10 vs. 7.18 ± 0.10, P > 0.05) (Table 3). In the antagonist group, 98 cycles had at least one early-cleavage embryo for transfer while the remaining 105 cycles had all late-cleavage embryos for transfer. There was no significant difference in pregnancy rate between the early-cleavage and late-cleavage subgroups (45.9% vs. 43.8%, P > 0.05) (Table 3).

**Table 3:** Embryo scores and mean numbers of blastomeres of the early-cleavage and late-cleavage subgroups in the antagonist group.

	Early-cleavage subgroup	Late-cleavage subgroup	P value
Day 2 embryo score	1.92 ± 0.04	2.11 ± 0.04	NS
No of day 2 blastomeres	4.11 ± 0.06	3.21 ± 0.06	< 0.05
Day 3 embryo score	2.02 ± 0.06	2.10 ± 0.06	NS
No. of day 3 blastomeres	7.29 ± 0.10	7.18 ± 0.10	NS
Pregnancy rate (%)	45.9 (45/98)	43.8 (46/105)	NS

Note: Values are the mean ± SEM. NS: no significance.

## Discussion

In the present study, we observed that embryonic early cleavage rate was significantly lower in the GnRH antagonist protocol compared with that in the GnRH long agonist protocol (27.7% vs. 41%, P < 0.0001). Although fewer oocytes were obtained in the GnRH antagonist groups, the mean numbers of normal fertilized oocytes, implantation rate, and ongoing pregnancy rate were all comparable with those in the GnRH agonist group. Consistent with previous studies [1-6], we also observed that embryonic early-cleavage was a good predictor for early embryonic development and pregnancy outcome in using a GnRH agonist long protocol (Table 2). However, these relationships were not found in using the GnRH antagonist protocol (Table 3).

To achieve a singleton pregnancy without reducing the implantation rate should be the primary goal in assisted reproduction treatment. Up to now, embryonic morphology has been one of the most useful tools to achieve this goal. In recent years, embryonic early-cleavage observed 25–27 hours after insemination has been suggested as another available parameter for embryo selection [1-6]. Moreover, all these previous studies had used a GnRH agonist long protocol for pituitary suppression. In the present study, we found that embryonic early-cleavage rate was significantly lower in using the GnRH antagonist protocol than the agonist protocol. In our previous study [13], we observed that the early-cleavage rate was significantly lower with the GnRH antagonists and was not a reliable predictor of pregnancy in women older than 35. In the present study, we also observed this relationship without any age limitation.

In the present study, the results showed that the early-cleavage embryos were of better quality and resulted in a higher pregnancy rate than the late-cleavage embryos in the GnRH agonist group. This was consistent with previous studies [1-6]. However, embryo quality and pregnancy rate were not significantly different between the early and late cleavage embryos in the GnRH antagonist group (Table 3). GnRH antagonists act through competitive binding to GnRH receptors, a situation that can be reversed by GnRH. Previous studies have shown that continuous exposure to GnRH antagonists will induce down-regulation of GnRH receptors and gene expression [15,16]. With the discovery of extra-pituitary GnRH receptors in reproductive tissues, including the uterus, endometrium, oocyte-cumulus complex, pre-implantation embryo and placenta [11,12,17], it is possible that the use of GnRH antagonists may lead to down-regulation of the receptors. Several previous studies using cancer cell lines have investigated the effect of GnRH antagonists at the cellular level. [18,19] In these in-vitro studies, GnRH antagonists interfered with cell growth by decreasing the synthesis of insulin-like growth factor (IGF) and inositol 1, 4, 5-trisphosphate (IP_3_). Another study showed that a decrease of epidermal growth factor (EGF) receptor mRNA and EGF peptide occurs in the presence of GnRH antagonists in vitro [20]. Both EGF and IGF receptors form the basis for induction of an important MAP kinase (MAPK)-mediated mitogenic cascade [21,22]. Based on the results of the above studies, theoretically, it is possible that the first mitosis of zygote (early cleavage) could be retarded if IGF, EGF and the MAPK signal transduction pathway are inhibited by GnRH antagonists. Another study showed the presence of GnRH receptor mRNA in the developing embryo and the pre-implantation embryonic development was significantly decreased by GnRH antagonists [11].

In IVF cycles, administration of GnRH antagonist is suspended once hCG is given, and the embryo is transferred between 5–7 days after the last dose of GnRH antagonist. The half-life of GnRH antagonist stated by the manufacturer is 30 hours and the time interval between the last dose of GnRH antagonist and the early cleavage of zygotes is about 75 to 90 hours. Consequently, the GnRH antagonist may still have some effects on delaying the first mitosis of zygotes. Moreover, there was also a significant difference in the day 2 mean numbers of blastomeres between the early-cleavage and late-cleavage embryos in the GnRH antagonist group (Table 3). However, in the following 24 hours the mean numbers of blastomeres on day 3 were not significantly different between the early-cleavage and late-cleavage embryos in the GnRH antagonist group (Table 3). Furthermore, the average embryo scores on days 2 and 3 were also not significantly different between the early-cleavage and late-cleavage embryos in the GnRH antagonist group (Table 3). Therefore, the effects of GnRH antagonist on the pre-implantation embryo may only be limited to the first 2 days after insemination or ICSI. The potential detrimental effects of GnRH antagonist upon the developing embryo may be significantly diminished 2 days after insemination. This could be the reason for a lack of any significant negative effect of GnRH antagonists on pregnancy and live birth outcome [9,10,23,24]. Based on the results in table 3 and previous reports, early cleavage of zygote is not a reliable predictor for pregnancy potential in using the GnRH antagonist protocol.

In the present study, we observed that using the GnRH antagonist protocol, the mean numbers of obtained oocytes and mature oocytes were significantly fewer than using the GnRH long agonist protocol. But the mean numbers of normal fertilized oocytes, pregnancy rate, implantation rate, and ongoing pregnancy rate were comparable between the two groups, which is in concordance with previous studies [25,26]. Though the GnRH antagonist protocol resulted in fewer oocytes, the pregnancy rate and normal fertilized embryos were comparable with the GnRH long agonist protocol. These findings might be due to decreased proportion of aneuploid and mosaic embryos in the GnRH antagonist protocol and the oocytes more likely to result in pregnancy were preferentially selected [25,26].

## Conclusion

In conclusion, the results of this study showed that the early-cleavage rate was significantly lower in the GnRH antagonist protocol than that in the GnRH long agonist protocol. Consistent with previous studies, the results of this study showed that the early-cleavage status of zygotes is indeed a parameter for embryo selection when using the GnRH agonist long protocol for ovarian stimulation. However, the results also showed that early cleavage of zygote seems not to be a powerful predictor for embryo implantation potential when the GnRH antagonist protocol was applied. Furthermore, despite lower numbers of oocytes were obtained in the GnRH antagonist protocol, it still showed comparable implantation rate to the GnRH agonist protocol.

## Competing interests

The authors declare that they have no competing interests.

## Authors' contributions

WJY carried out the data collection, performed the statistical analysis and drafted the manuscript. YMH participated in the design of the study and helped to draft the manuscript. RKKL carried out the data collection and supervised the analysis. SHL carried out semen analysis and statistical analysis. SF helped in the study coordination and drafted the manuscript. All authors read and approved the final manuscript.
